# AAV-Syn-BDNF-EGFP Virus Construct Exerts Neuroprotective Action on the Hippocampal Neural Network during Hypoxia In Vitro

**DOI:** 10.3390/ijms19082295

**Published:** 2018-08-05

**Authors:** Elena V. Mitroshina, Tatiana A. Mishchenko, Alexandra V. Usenko, Ekaterina A. Epifanova, Roman S. Yarkov, Maria S. Gavrish, Alexey A. Babaev, Maria V. Vedunova

**Affiliations:** 1 Lobachevsky State University of Nizhni Novgorod, Institute of Neuroscience, 23 Prospekt Gagarina, 603950 Nizhny Novgorod, Russia; saHarnova87@mail.ru (T.A.M.); sas7342415@mail.ru (A.V.U.); epifa888@mail.ru (E.A.E.); roman.sultanov.96@mail.ru (R.S.Y.); gavrish@neuro.nnov.ru (M.S.G.); alexisbabaev@list.ru (A.A.B.); MVedunova@yandex.ru (M.V.V.); 2 Privolzhskiy Research Medical University, 10/1 Minin and Pozharsky Square, 603005 Nizhny Novgorod, Russia

**Keywords:** adeno-associated virus vector, brain-derived neurotrophic factor, BDNF, cerebral hypoxia, primary hippocampal cell cultures, multielectrode arrays, calcium imaging, neuroprotection

## Abstract

Brain-derived neurotrophic factor (BDNF) is one of the key signaling molecules that supports the viability of neural cells in various brain pathologies, and can be considered a potential therapeutic agent. However, several methodological difficulties, such as overcoming the blood–brain barrier and the short half-life period, challenge the potential use of BDNF in clinical practice. Gene therapy could overcome these limitations. Investigating the influence of viral vectors on the neural network level is of particular interest because viral overexpression affects different aspects of cell metabolism and interactions between neurons. The present work aimed to investigate the influence of the adeno-associated virus (AAV)-Syn-BDNF-EGFP virus construct on neural network activity parameters in an acute hypobaric hypoxia model in vitro. Materials and methods. An adeno-associated virus vector carrying the *BDNF* gene was constructed using the following plasmids: AAV-Syn-EGFP, pDP5, DJvector, and pHelper. The developed virus vector was then tested on primary hippocampal cultures obtained from C57BL/6 mouse embryos (E18). Acute hypobaric hypoxia was induced on day 21 in vitro. Spontaneous bioelectrical and calcium activity of neural networks in primary cultures and viability tests were analysed during normoxia and during the posthypoxic period. Results. BDNF overexpression by AAV-Syn-BDNF-EGFP does not affect cell viability or the main parameters of spontaneous bioelectrical activity in normoxia. Application of the developed virus construct partially eliminates the negative hypoxic consequences by preserving cell viability and maintaining spontaneous bioelectrical activity in the cultures. Moreover, the internal functional structure, including the activation pattern of network bursts, the number of hubs, and the number of connections within network elements, is also partially preserved. BDNF overexpression prevents a decrease in the number of cells exhibiting calcium activity and maintains the frequency of calcium oscillations. Conclusion. This study revealed the pronounced antihypoxic and neuroprotective effects of AAV-Syn-BDNF-EGFP virus transduction in an acute normobaric hypoxia model.

## 1. Introduction

Currently, there is a significant amount of experimental data suggesting that brain-derived neurotrophic factor (BDNF) is an effective neuroprotective agent for various central nervous system (CNS) diseases and brain injuries. Both in vitro [1,2,3] and in vivo [4,5,6,7] studies show its role as a corrector of the consequences of individual ischaemic factors influence (e.g., hypoxia) as ischaemia at all. For instance, BDNF application reduces the volume of cerebral infarction area in the middle cerebral artery occlusion (MCAo) in rodents [8,9,10]. Studies conducted in other animal ischaemia models also showed that BDNF minimizes cell death, decreases the volume of the site of stroke, and accelerates the restoration of neurological status and cognitive abilities [11,12,13]. Moreover, extensive experimental data indicate that BDNF plays an important role in the pathogenesis of neurodegenerative diseases. It has been shown that the expression of BDNF mRNA and the level of mature BDNF significantly decreases in the hippocampus and the cerebral cortex during the development of Alzheimer’s disease [14,15,16]. In the case of recombinant BDNF application in Alzheimer’s disease [17,18,19] and Parkinson’s disease [4,20,21], significant inhibition of neurodegenerative processes in the brain were observed.

As one of the key representatives of the neurotrophin family, BDNF promotes neuronal growth and brain development in embryogenesis and is involved in the formation and maintenance of neural network functioning in the postnatal period [22,23]. Moreover, BDNF actively participates in the modulation of synaptic plasticity in the hippocampus and in several parts of the cerebral cortex [24,25,26]. A number of studies have also shown that the use of BDNF can stimulate neurogenesis and the migration of neuronal progenitors from the subventricular zone to the site of stroke [27,28,29]. The important feature of BDNF in maintaining the viability of neurons that are not included in the neural network structure in early ontogenesis [4,30,31,32] suggests the possibility of using this neurotrophin for the long-term maintenance of the viability of neurons that have lost their connections with the neural network due to destruction in the post-ischaemic period.

To date, a number of research groups are working on elaborating and testing neurotrophin therapeutic delivery systems, such as nanoparticulate carriers [33,34,35,36] and viral vector constructs for long-term BDNF overexpression in nervous system cells [37,38,39,40]. This is a promising method for ischaemia therapy, as it may avoid the need for prolonged use of drugs and have targeted effects on a strictly defined cell type. It was recently shown that intraventricular administration of adeno-associated virus (AAV), carrying the *BDNF* gene, reduces the site of cerebral infarction [41]. Yu et al. demonstrated the acceleration of locomotor function recovery and migration of neurons from the subventricular zone to the affected hemisphere in a rat stroke model with AAV-BDNF application [38].

However, detailed investigations describing the effect of BDNF overexpression on neural network activity under the influence of various damaging factors have not been previously conducted. Therefore, our present study is devoted to investigating the possible neuroprotective effect of BDNF overexpression activated by AAV-BDNF on the functional bioelectrical and calcium activity of neural networks in primary hippocampal cultures in a hypoxia model in vitro.

## 2. Results

### 2.1. Development and Testing of the AAV-Syn-BDNF-EGFP Vector on Primary Hippocampal Cell Cultures

To increase BDNF expression in brain neurons, an adeno-associated virus vector carrying the *BDNF* gene was constructed at the first stage of the study. For BDNF amplification, we selected a system of primers, mBDNF-EcoRI-fw and mBDNF-BamHI-rv (5′-ATTGAATTCATGGGCCACATGCTGTCC-3′ and 5′-AATGGATCCAATCTTCCCCTTTTAATGGTCAGTG-3′) containing restriction sites for the subsequent restriction and ligation into the constructed vector, temperature regimes, and the time of the reaction. The plasmid isolated from *E. coli* was tested for the correct assembly by a restriction reaction and polymerase chain reaction (PCR) on the cloned fragment of the *BDNF* gene. The lengths of the obtained fragments were then electrophoretically verified (Figure S1).

**Figure 1:**
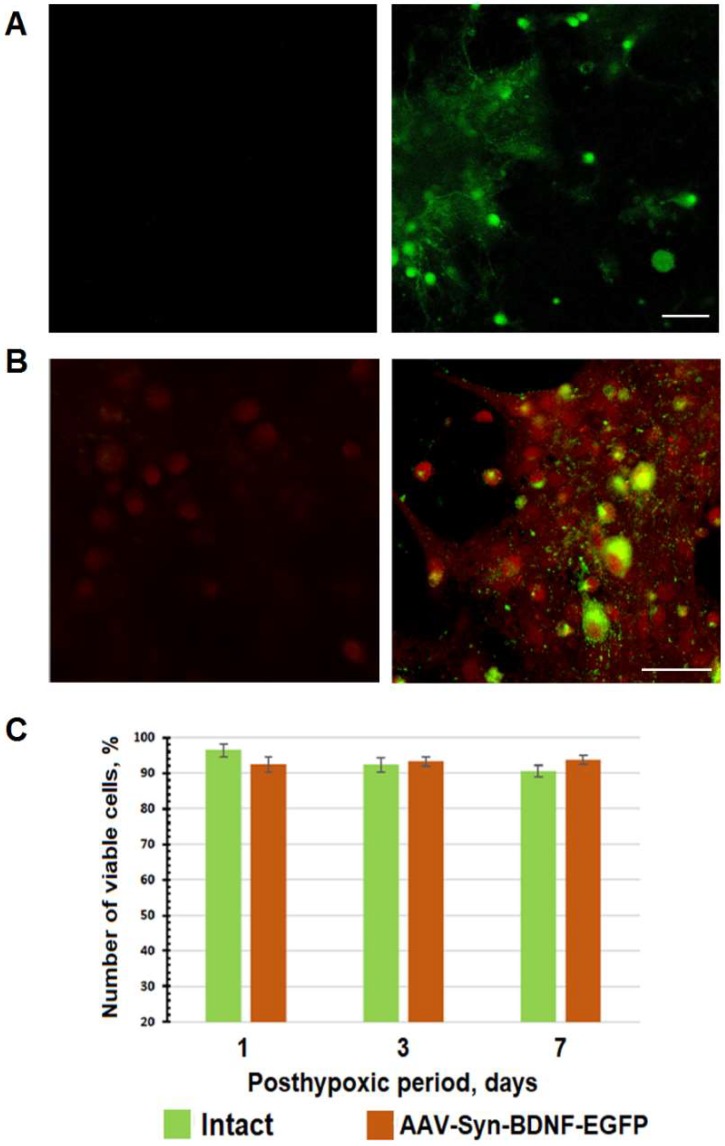
In vitro testing of the adeno-associated virus (AAV)-Syn-brain-derived neurotrophic factor (BDNF)-EGFP virus vector. (**А**) EGFP expression in neurons day 7 after transduction (left column—control; right column—cells transduced by AAV-Syn-BDNF-EGFP). Scale bar: 50 μm; (**B**)Immunocytochemical staining of BDNF protein in primary hippocampal cultures on day 7 after AAV-Syn-BDNF-EGFP transduction (left column—sham; right column—culture transduced by AAV-Syn-BDNF-EGFP). Red channel—BDNF-positive cells; green channel—fluorescence of EGFP. Scale bar: 20 μm. Transduction by AAV-Syn-BDNF-EGFP virus vector increases BDNF expression in primary hippocampal cultures; (**С**) Analysis of cell viability in primary hippocampal cell cultures for 7 days after AAV-Syn-BDNF-EGFP transduction, analysis of variance (ANOVA), *n* = 18. The viability of transduced cells does not differ from that of nontransduced cells.

Next, the developed AAV-Syn-BDNF-EGFP vector was tested on primary hippocampal cell cultures. Control of the expression of green fluorescent protein EGFP was performed from the first day after transduction using confocal microscopy. EGFP expression was observed beginning on day 4 after transduction for the following three weeks (Figure 1A). Immunocytochemical staining with monoclonal antibodies against BDNF showed that application of the developed virus vector increased BDNF production in primary hippocampal cells by 2.7 times (Figure 1B).

As a result of the high sensitivity of the neuronal cultures to almost all chemical treatments, including viral drugs, the developed virus construct was examined for possible cytotoxic effects. Therefore, we investigated the viability of primary hippocampal cell cultures on day 7 after virus vector infection. The experiments on the cytotoxicity of AAV-Syn-BDNF-EGFP did not reveal any changes in the viability of neural networks in primary hippocampal cultures (Figure 1C). 

### 2.2. The Influence of the AAV-Syn-BDNF-EGFP Vector on Spontaneous Bioelectrical Activity in Primary Hippocampal Cell Cultures

Hereinafter, we investigated the influence of the developed AAV-Syn-BDNF-EGFP virus vector on the spontaneous bioelectrical activity of neural networks in primary hippocampal cultures. Because it was previously shown that a single BDNF (1 ng/mL) administration was able to modulate the spontaneous bioelectrical activity of primary hippocampal cultures [24,42], we decided to investigate the effect of chronic BDNF overexpression on spontaneous bioelectrical activity and the functional structure of neural networks under normal conditions. To achieve this goal, AAV-Syn-BDNF-EGFP transduction of primary hippocampal cultures was performed on the seventh day in vitro (DIV). Ten-minute recordings of spontaneous bioelectrical activity at constant conditions (35.5 °C, 5% CO_2_) were recorded daily until 21 DIV.

Recently, alterations of the activity pattern and the appearance of a specific stable pattern of network bursts during primary culture development were demonstrated. These observations were associated with the formation of a stable structure of the neural network with a maximum possible number of synapses in the absence of afferentation of external signals. The formation of stable, regularly repeated bursts occurred by 8–10 DIV. Further culture development (14 DIV) was characterized by the appearance of normal inverted and then noninverted bursts, accompanied by a gradual increase in the number of spikes in a burst, which persisted until 19–21 DIV [43]. 

**Figure 2:**
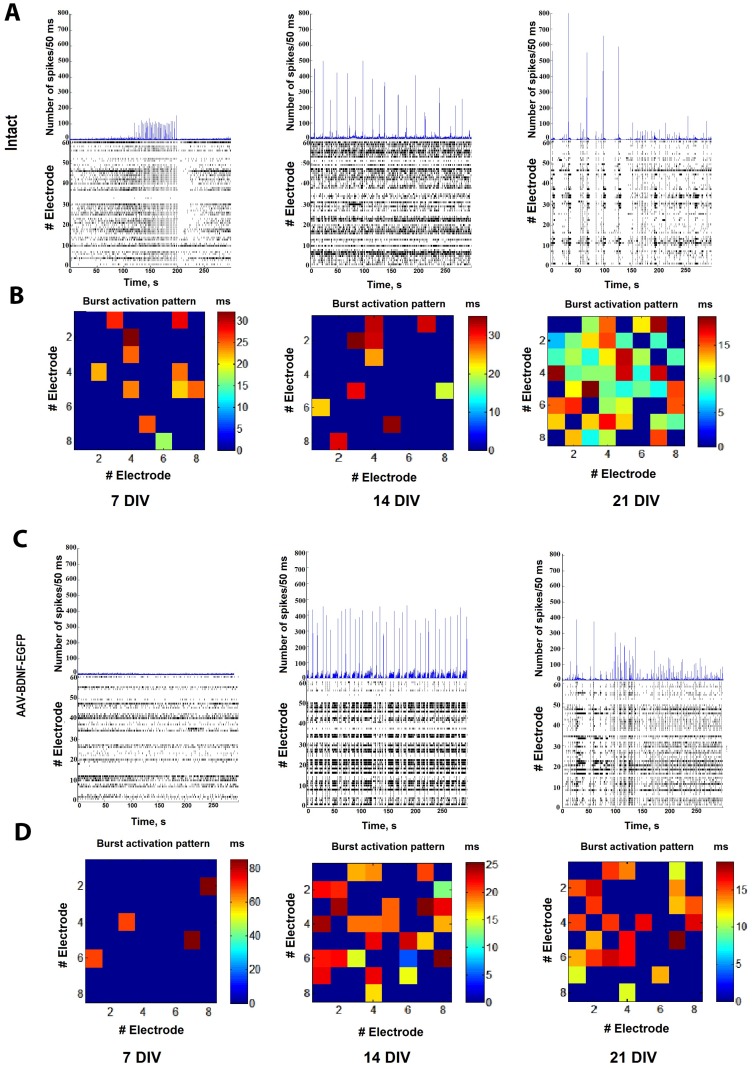
Spontaneous bioelectrical activity alterations in primary hippocampal cell cultures on 7, 14 and 21 days in vitro (DIV). (**A**,**C**) Number of spikes/50 ms and raster diagrams of spontaneous bioelectrical activity in primary hippocampal cultures: (**А**) sham, (**C**) AAV-Syn-BDNF-EGFP; (**B**,**D**) representative examples of the activation pattern of spontaneous bioelectrical activity in primary hippocampal cultures: (**B**) sham, (**D**) AAV-Syn-BDNF-EGFP. The colour scale corresponds to the time of occurrence of the first spike in the network burst and is presented in squares according to the electrodes of the multi-electrode array. The data show the absence of changes in the main parameters of spontaneous bioelectrical activity in primary culture with BDNF overexpression compared with sham cultures. Changes in the activation pattern were detected on 21 DIV.

An analysis of the main parameters of spontaneous bioelectrical activity did not reveal any significant changes in the response to BDNF overexpression by AAV-Syn-BDNF-EGFP from 8 DIV (1st day after transduction) until 21 DIV (14th day after transduction), including the number of small network bursts/10 min (8 DIV-sham: 56.6 ± 4.9, AAV-Syn-BDNF-EGFP: 63.2 ± 10.56; 14 DIV—sham: 102.2 ± 18.6, AAV-Syn-BDNF-EGFP: 135.2 ± 24.8; 21 DIV—sham: 186 ± 23.5, AAV-Syn-BDNF-EGFP: 235.5 ± 43.15) and the number of spikes in a burst (8 DIV—sham: 224.2 ± 30.9, AAV-Syn-BDNF-EGFP: 183.2 ± 30.6; 14 DIV—sham: 302.2 ± 18.6, AAV-Syn-BDNF-EGFP: 310.2 ± 20.8, 21 DIV—sham: 483 ± 52.5, AAV-Syn-BDNF-EGFP: 438.5 ± 61.5, *p* < 0.05, ANOVA, N = 6). Notably, the number of spikes in a burst tended to increase in AAV-Syn-BDNF-EGFP-infected cultures compared with intact cultures at 13–15 DIV (Figure 2; Table 1).

Moreover, infection with the developed virus vector led to changes in the activation pattern of the neural network bursts on 21 DIV (Figure 2B,D). We found that the time of signal propagation through the network tended to increase in primary cultures transduced by AAV-Syn-BDNF-EGFP (time of occurrence of the first spike in the network burst: sham: 5–10 ms; AAV-Syn-BDNF-EGFP: 10–15 ms)

Thus, the BDNF overexpression induced by AAV-Syn-BDNF-EGFP does not exert a significant influence on the spontaneous bioelectrical activity of the neural networks in primary hippocampal cultures in comparison with that of intact cultures. However, increasing BDNF overexpression affects the burst activation pattern, causing a change in the time of signal propagation over the network. Our findings suggest that AAV-Syn-BDNF-EGFP-induced BDNF overexpression does not have pronounced neurotropic activity, but is able to influence the functional structure of the neural network.

### 2.3. Effects of the AAV-Syn-BDNF-EGFP Vector on the Functional Structure of Neural Networks in Primary Hippocampal Cell Cultures

**Figure 3:**
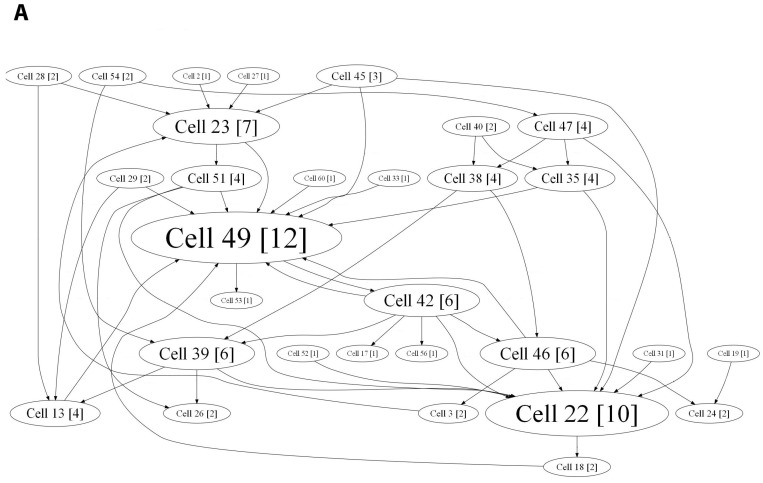

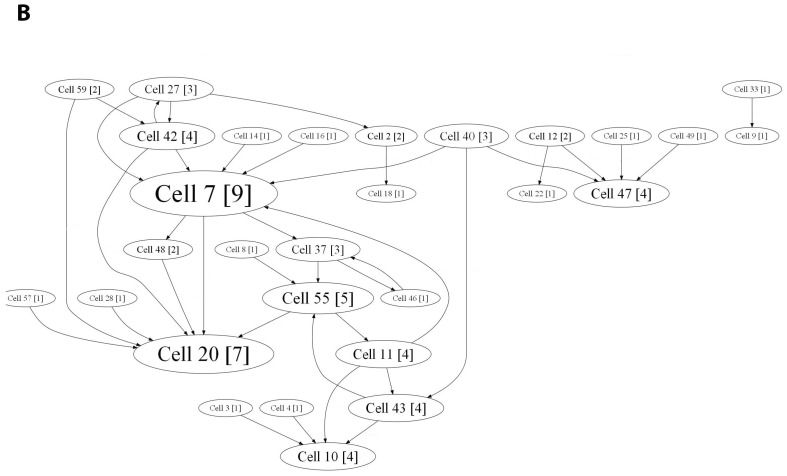
Internal functional structure of neural networks in primary hippocampal cultures after AAV-BDNF-EGFP transduction. Graphical representation of the correlated connections among neurons in the network on 14 DIV. The electrode number is presented as “Cell X”. The number of connections on the electrode is indicated in square brackets. The vertex size is proportional to the number of significant connections. **А**—sham, **B**—AAV-Syn-BDNF-EGFP.

**Figure 4:**
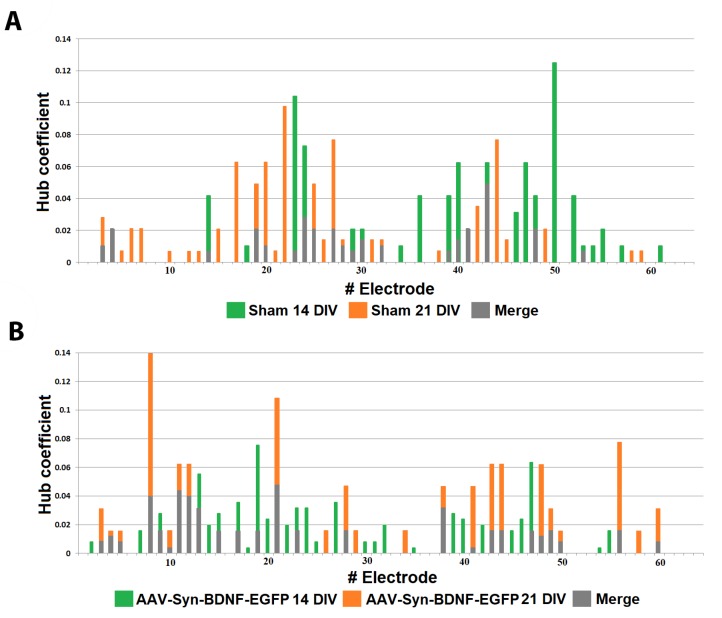
Functional rearrangement alterations of neural networks during the 14–21 DIV period. The hub coefficient was calculated as the ratio of the number of connections of an electrode to its total number in the graph and therefore characterizes the importance of a group of neurons located at one electrode for network activity. Plotting the hub coefficients allows estimation of the changes in the significance of each electrode in a multi-electrode array. **A**—sham; **B**—AAV-Syn-BDNF-EGFP.

For a more detailed analysis of the network effects accompanied by BDNF overexpression and to determine the internal neural network structure, we applied a cross-correlation method and graphs. This method allows the detection of hubs, that is, the elements with the maximum number of functionally active connections, and shows the dynamic changes occurring in the network in short- and long-term periods. The hub coefficient was calculated as the ratio of the number of connections of an electrode to its total number in the graph; therefore, it characterizes the importance of a group of neurons located at one electrode for network activity. Plotting the hub coefficient allows estimation of the changes in the significance of each electrode in a multi-electrode array. Examination of the neural network bursts revealed that the cultures in both the intact and AAV-Syn-BDNF-EGFP groups have constant hubs in their functional network structure at different stages of development in vitro (7–21 DIV). During this period, the neural network structure became more complicated. At 21 DIV, the number of hubs was 4.75 ± 0.959 in the “sham” group and 3.81 ± 0.51 in the “AAV-Syn-BDNF-EGFP” group (Figure 3), with 13.67 ± 0.97 and 13.14 ± 1.28 connections per hub, respectively. Thus, application of the developed virus vector did not influence the main parameters of the neural network functional structure. Notably, a slight redistribution of hubs was observed during the development of the cultures in vitro, with the percentage of overlap between 14 DIV and 21 DIV being 40.65 ± 11.19% in the “sham” group and 45.07 ± 12.76% in the “AAV-Syn-BDNF-EGFP” group (Figure 4). 

Thus, BDNF overexpression induced by AAV-BDNF-EGFP does not significantly rearrange the neural network in normal conditions. This positive argument points to the potential use of the developed virus vector as a therapeutic agent.

### 2.4. Neuroprotective Action of the AAV-Syn-BDNF-EGFP Vector in the Hypoxia Model In Vitro

The next stage of the study was devoted to comprehensively investigating the antihypoxic and neuroprotective effects of BDNF overexpression under an acute hypoxic state. To compare the effects of different methods of BDNF administration, recombinant BDNF (1 ng/mL) was applied daily from 7 DIV.

Analysis of cell viability in primary hippocampal cultures during the posthypoxic period revealed a significant (*p* < 0.05, ANOVA) increase in the number of dead cells. On day 3 of the posthypoxic period, the percentage of viable cells in the “hypoxia” group was 91.23 ± 0.22%, whereas the percentage of viable cells in the “sham”, “hypoxia + BDNF”, and “hypoxia + AAV-Syn-BDNF-EGFP” groups of cultures was significantly higher (*р* < 0.05, ANOVA), accounting for 98.07 ± 0.58%, 97.21 ± 0.43%, and 96.28 ± 0.58%, respectively (Table 2). 

The following percentages of viable cells were observed on day 7 of the posthypoxic period (28 DIV): “sham”: 91.39 ± 1.34%; “hypoxia”: 70.46 ± 5.14%; “hypoxia + BDNF”: 86.86 ± 4.57%; “hypoxia + AAV-Syn-BDNF-EGFP”: 91.75 ± 1.51%. Thus, both examined methods of BDNF administration showed pronounced neuroprotective effects. There was no significant difference between the “hypoxia + BDNF” and “hypoxia + AAV-Syn-BDNF-EGFP” groups. Thus, the observed neuroprotective effect of the developed virus construct was consistent with our initial expectations and literature data [2,3,44].

Next, we investigated the influence of AAV-hSyn-BDNF-EGFP virus transduction on the spontaneous bioelectrical activity of primary hippocampal cultures in a hypoxic state. 

**Figure 5:**
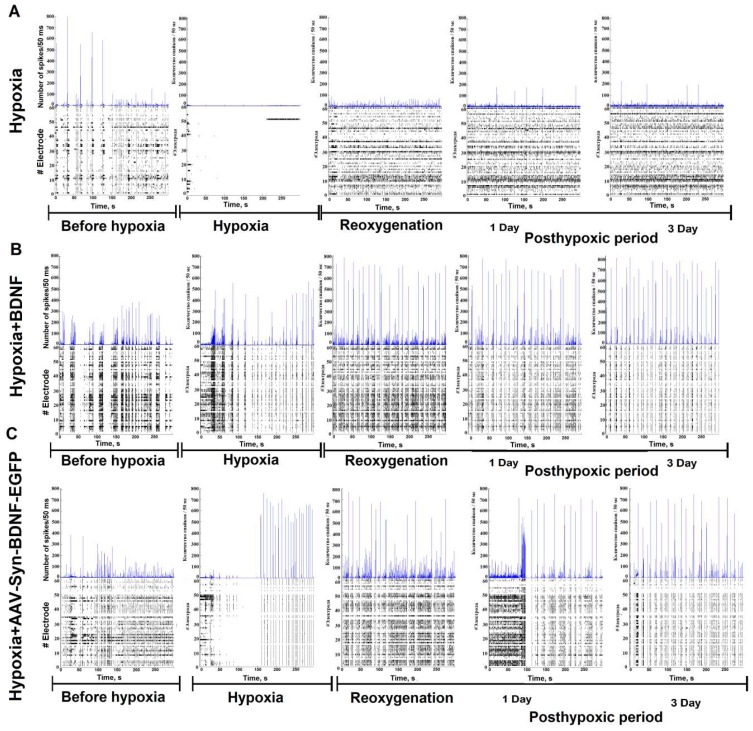
Number of spikes/50 ms and raster diagrams of spontaneous bioelectrical activity in primary hippocampal cultures during hypoxia and in the posthypoxic period: **А**—hypoxia, **B**—hypoxia + BDNF, **C**—hypoxia + AAV-Syn-BDNF-EGFP.

Acute oxygen deprivation reduced the spontaneous bioelectrical activity of primary hippocampal cultures, consistent with previous reports [2,45]. Spontaneous bioelectrical activity was suppressed in the “hypoxia” group from the 3rd minute of acute oxygen deficiency and was restored in the reoxygenation period. The number of network bursts did not differ between the baseline and 2 h after reoxygenation (number of small network bursts/10 min: before hypoxia: 186 ± 23.5; hypoxia: 2.20 ± 1.78; reoxygenation: 251.5 ± 48.35). However, the number of spikes in a burst decreased considerably (before hypoxia: 483 ± 52.5; hypoxia: 15.00 ± 3.81; reoxygenation: 64.5 ± 18.51) (Figure 5). In the “hypoxia + BDNF” and “hypoxia + AAV-hSyn-BDNF-EGFP” groups, neural network burst activity was maintained at the baseline level during all observation periods (number of small network bursts/10 min- “hypoxia + BDNF”: before hypoxia: 203.5 ± 29.15; hypoxia: 195.5 ± 42.04; reoxygenation: 198.2 ± 34.65; “hypoxia + AAV-Syn-BDNF-EGFP”: before hypoxia: 235.5 ± 43.15; hypoxia: 169.00 ± 47.00; reoxygenation: 215.9 ± 41.56, ANOVA, *n* = 4). Of note, in the “hypoxia” group, the number of spikes per burst during the reoxygenation period was reduced by 7.54 times, while BDNF application maintained this parameter at the baseline level (number of spikes in a burst – “hypoxia + BDNF”: before hypoxia: 383.5 ± 39.75; hypoxia: 350.5 ± 43.64; reoxygenation: 555.5 ± 43.64; “hypoxia + AAV-Syn-BDNF-EGFP”: before hypoxia: 438.5 ± 61.5; hypoxia: 479.00 ± 37.15; reoxygenation: 615.9 ± 64.56, ANOVA, *n* = 4).

Both the application of recombinant BDNF and AAV-Syn-BDNF-EGFP contributes to maintaining key parameters of spontaneous bioelectrical activity in the posthypoxic period. Compared with baseline, in the “hypoxia” group, the number of network bursts and the number of spikes in a burst decreased by 1.6 and 1.4 times the day after hypoxia and by 3.1 and 4.8 times, respectively, on day 7 of the posthypoxic period. The day after oxygen deficiency, in the “hypoxia + BDNF”, the number of network bursts did not differ from baseline, whereas the number of spikes in a burst increased by 1.9 times. On day 7 of the posthypoxic period, the number of network bursts and spikes in a burst were decreased by 2.5 and 2.1 times, respectively, compared with the baseline.

Similar observations were found in the “hypoxia + AAV-BDNF-EGFP”. The day after hypoxia modelling, the number of network bursts was comparable to that of baseline, whereas the number of spikes in a burst increased by 2.6 times. On day 7 of the posthypoxic period, the number of bursts was decreased by 2.1 times, the total amount of spikes remained heightened, and the baseline level increased by 1.8 times.

**Figure 6:**
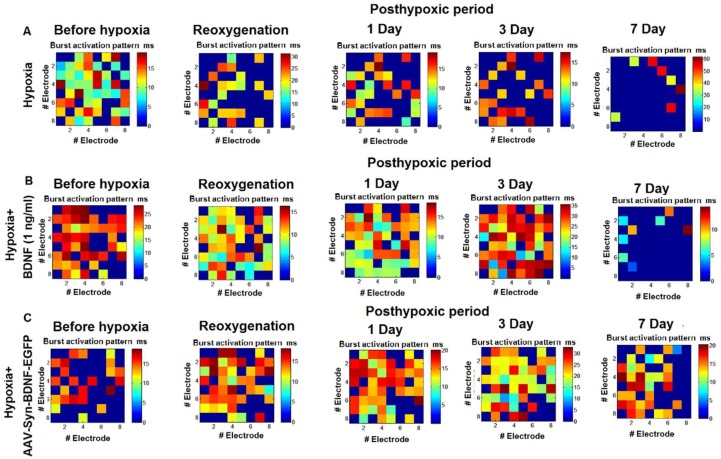
Representative examples of the activation pattern of spontaneous bioelectrical activity in primary hippocampal cultures in the posthypoxic period: **A**—hypoxia, **B**—hypoxia+BDNF (1 ng/mL), **C**—hypoxia + AAV-Syn-BDNF-EGFP. The colour scale corresponds to the time of occurrence of the first spike in the network burst and is presented in squares according to the electrodes of the multielectrode array. The data show that hypoxia induced a decrease in the signal transmission speed in the network and significant changes in the activation pattern of the network burst. BDNF overexpression contributed to maintaining the activation pattern in the posthypoxic period.

An analysis of the activation pattern in the “hypoxia” group revealed a significant decrease in the signal transmission speed in the network by day 7 of the posthypoxic period (Figure 6). These observations are connected with a great number of neuronal deaths within the network. BDNF overexpression preserved the functional structure of the network burst and maintained the time of signal transmission at the initial level.

### 2.5. The Influence of the AAV-Syn-BDNF-EGFP Vector on the Functional Structure of Neural Networks in the Modelled Hypoxic State

**Figure 7:**
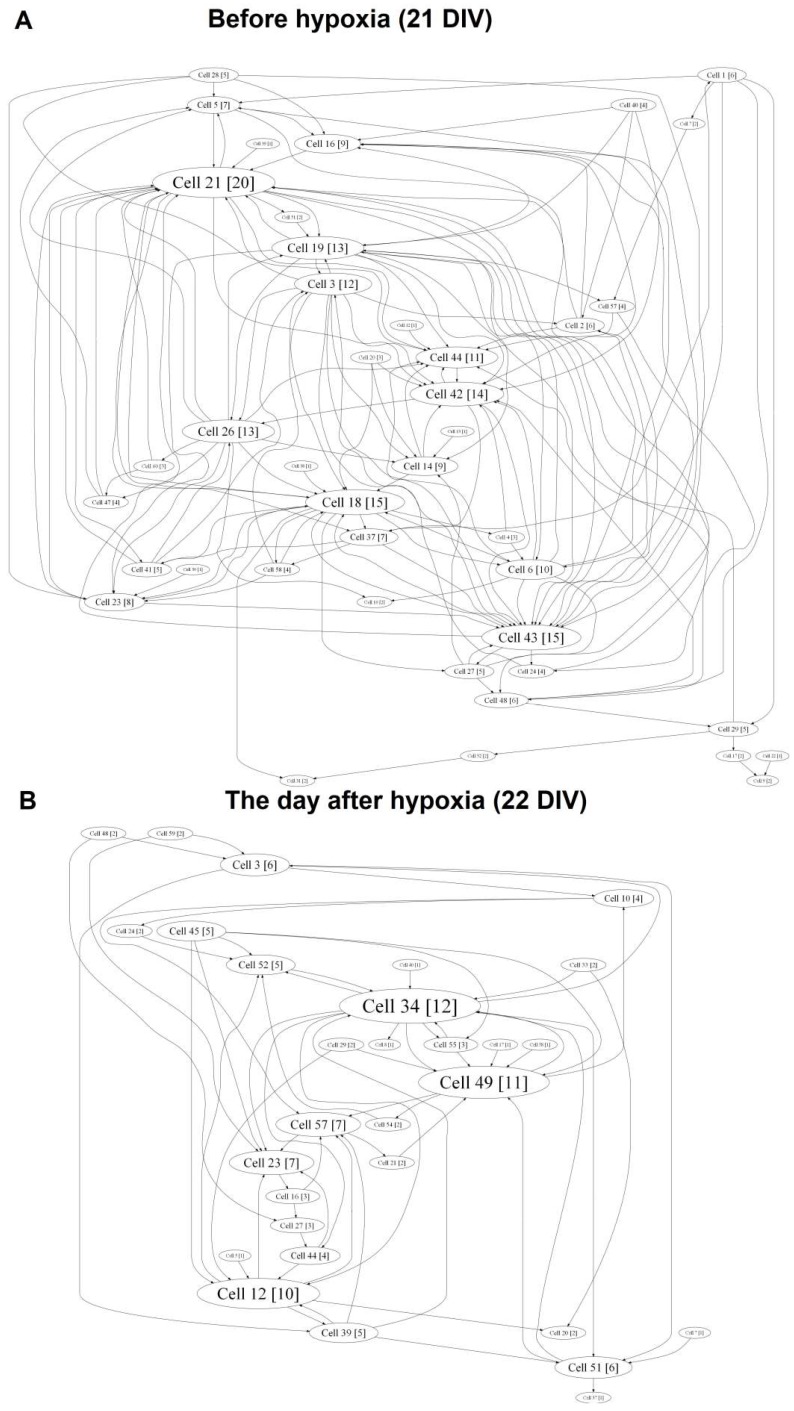
Internal functional structure of neural networks in the primary hippocampal cultures in hypoxia modelling. Graphical representation of the correlated connections among neurons in the network. The electrode number is presented as “Cell X”. The number of connections on the electrode is indicated in square brackets. The vertex size is proportional to the number of significant connections. **A**—before hypoxia (21 DIV), **B**—the day after hypoxia.

Cross-correlation analysis established that acute hypoxia induced a simplified internal neural network functional structure in primary hippocampal cultures and reduced the number of hubs beginning on the first day after hypoxia induction (before hypoxia: 4.75 ± 0.96; day after hypoxia: 3.5 ± 0.87) (Figure 7).

**Figure 8:**
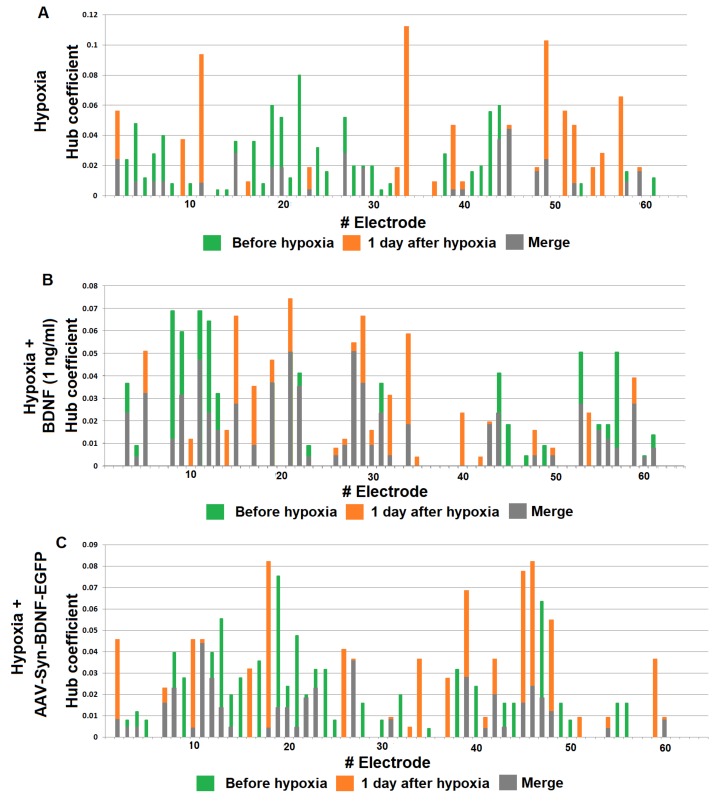
Functional rearrangement alterations of neural networks in the posthypoxic period. The hub coefficient was calculated as the ratio of the number of connections of an electrode to its total number in the graph, and thus characterizes the importance of a group of neurons located at one electrode for network activity. Plotting the hub coefficient allows estimation of the changes in the significance of each electrode in a multi-electrode array. (**A**) Hypoxia; (**B**) hypoxia + BDNF 1 ng/mL (**C**) hypoxia + AAV-Syn-BDNF-EGFP. The data show changes in the significance of individual electrodes in the “hypoxia” group on day 1 of the posthypoxic period (**A**), whereas the hypoxia + BDNF and hypoxia + AAV-Syn-BDNF-EGFP groups (**B**,**C**) had a preserved number of main hubs in the network, although some displacement of several individual electrodes was observed.

The hub coefficient, which characterizes the importance of a group of neurons on the electrode for network activity, revealed a significant (*p* < 0.05, ANOVA) decrease in the number of connections to active electrodes in the “hypoxia” group (hub coefficient: before hypoxia: 0.023 ± 0.003, the day after hypoxia: 0.032 ± 0.005; the average number of connections in the hub: before hypoxia: 13.67 ± 0.97, the day after hypoxia: 11.00 ± 0.57). Moreover, the significance of individual electrodes was changed. The percentage of overlap in the “hypoxia” group was 41.09 ± 10.96% (Figure 8).

Both variants of BDNF application partially eliminate hypoxic effects on the neural network internal structure (Figures S2 and S3). In the “hypoxia + BDNF” and “hypoxia + AAV-BDNF-EGFP” groups, a comprehensive network structure without pronounced dominants, with several key hubs, was preserved. Moreover, the number of hubs was increased (number of hubs: “hypoxia + BDNF”: before hypoxia: 6.11 ± 0.52; the day after hypoxia: 7.88 ± 0.35; “hypoxia + AAV-BDNF-EGFP”: before hypoxia: 3.81 ± 0.96; the day after hypoxia: 7.45 ± 0.67; the average number of connections in the hub: “hypoxia + BDNF”: before hypoxia: 12.63 ± 0.65, the day after hypoxia: 14.33 ± 0.97; “hypoxia + AAV-BDNF-EGFP”: before hypoxia: 13.14 ± 1.28, the day after hypoxia: 13.75 ± 1.29). 

During displacement of the significance of several individual electrodes, the percentage of overlap in the “hypoxia + BDNF” and “hypoxia + AAV-BDNF-EGFP” groups was significantly higher than that in the “hypoxia” group (percentage of overlap: “hypoxia”: 41.09 ± 10.96%; “hypoxia + BDNF”: 74.96 ± 15.43%; “hypoxia + AAV-BDNF-EGFP”: 75.79 ± 15%, *p* < 0.05, ANOVA).

Thus, the developed virus vector partially eliminates the negative consequences of acute hypoxia, maintaining the spontaneous bioelectrical activity of primary hippocampal cultures and partially preserving the neural network functional structure, including the activation pattern and the main dominant hubs.

### 2.6. The Influence of the AAV-Syn-BDNF-EGFP Vector on Spontaneous Calcium Activity in Primary Hippocampal Cell Cultures in the Modelled Hypoxia In Vitro

In addition to electrophysiological studies, calcium dynamics are extremely indicative of the in-depth functional state of neural networks. Ca^2+^ ions play a special role in signal transmission. Changes in Ca^2+^ concentrations can be a “trigger” for activating many events in neural cells, such as exocytosis of synaptic vesicles, realization of different biochemical signaling mechanisms upon postsynaptic receptor activation, and so on. Therefore, the change in the intracellular concentration of Ca^2+^ ions can serve as a reliable parameter of functional activity in neuron-glial networks. To investigate the spatial distribution and fluctuations in Ca^2+^ concentration in cells, optical fluorescent calcium imaging was applied.

The several stages of the formation of calcium activity were previously established in primary hippocampal cultures. First, calcium events appeared on 5 DIV, and the percentage of cells that exhibited Ca^2+^ activity was less than 5%. Moreover, such activity was not synchronized. Rare (0.03 ± 0.007 Hz) and long-lasting (8 ± 0.55 s) Ca^2+^ oscillations appeared in the neuronal cultures by 7 DIV. At this stage of culture development, characterized by the appearance of the first mature chemical synapses, there was a tendency for Ca^2+^ activity synchronization, and the percentage of working cells was 5–10%. The total amount of cells exhibiting Ca^2+^ activity gradually increased from 14 DIV (50–70%) and reached 97% by 21 DIV [46].

**Figure 9:**
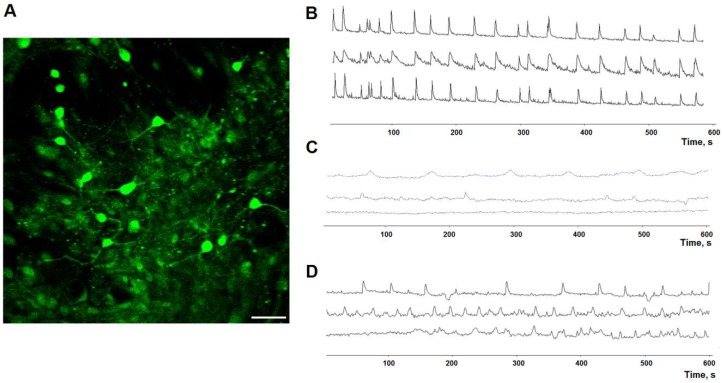
Ca^2+^ activity in primary hippocampal cultures on day 7 of the posthypoxic period. (**A**) Example fluorescence image of the calcium-sensitive dye Oregon green 488 BAPTA in culture. Scale bar 20 μm. (**B**–**D**) Representative Ca^2+^ recordings, (**B**) sham; (**C**) hypoxia; (**D**) hypoxia + AAV-BDNF-EGFP.

Evaluation of calcium activity in the primary hippocampal cultures transduced by AAV-Syn-BDNF-EGFP was performed on day 7 of the posthypoxic period. We showed that in the “hypoxia” group, the percentage of cells exhibiting Ca^2+^ activity was significantly decreased by 2.7 times compared with the “sham” group (“sham”: 97.66 ± 1.47%; “hypoxia”: 35.58 ± 5.46%). This parameter was significantly higher in the “hypoxia + AAV-BDNF-EGFP” group (65.14 ± 5.27%) than in the “hypoxia” group (Table 3, Figure 9).

The main parameters that characterize calcium events are duration and frequency. We revealed that hypoxia significantly decreased the frequency of Ca^2+^ oscillations by 5.7 times compared with the “sham” group (“sham”: 6.9 ± 0.31 osc/min; “hypoxia”: 1.24 ± 0.134 osc/min, *p* < 0.05, ANOVA). These findings are possibly connected to violations in the morpho-functional structure of primary hippocampal cultures and the massive neuronal death induced by hypoxia. The use of the AAV-BDNF-EGFP virus vector better preserved the frequency of Ca^2+^ oscillations in primary hippocampal cultures than in the “hypoxia” group (“hypoxia + AAV-BDNF-EGFP”: 5.84 ± 0.28 osc/min, *p* < 0.05, ANOVA).

Hypoxia significantly increased the duration of Ca^2+^ oscillations, whereas this parameter did not differ between the “hypoxia + AAV-BDNF-EGFP” and “sham” cultures (“sham”: 6.23 ± 0.16 s; “hypoxia”: 7.17 ± 0.28 s; “hypoxia + AAV-BDNF-EGFP”: 5.87 ± 0.09 s, *p* < 0.05, ANOVA).

Thus, data on spontaneous bioelectrical and calcium activity demonstrated that BDNF overexpression induced by AAV-Syn-BDNF-EGFP contributes to maintaining the functional activity of cells in primary hippocampal cultures exposed to acute hypoxia.

## 3. Discussion

BDNF is an important signaling molecule that contributes to maintaining functional integrity and neuronal survival in stress conditions [44,47]. Numerous experimental studies have indicated the effectiveness of BDNF against various brain pathologies, including hypoxic states [10,47,48] and neurodegenerative diseases [4,15,16,17,18,19,20,21]. Therefore, this neurotrophin could be considered a potential therapeutic agent for some of the most comprehensive pathologies associated with death and patient disability. However, several methodological difficulties, including overcoming the blood–brain barrier and the short half-life and excretion of the active molecules, challenge the potential use of BDNF in clinical practice. Gene therapy with viral vectors can overcome these limitations and provide sustained BDNF overexpression in tissues where it is necessary to maintain neuronal survival.

Our in vitro studies showed the antihypoxic and neuroprotective effects of an adenoviral vector containing the *BDNF* gene on primary hippocampal cultures in acute hypoxia modelling. Notably, we examined not only cell viability, but also the functionality of neural networks, including spontaneous bioelectrical and calcium activity, under the influence of hypoxia. Current publications on the neuroprotective effects of viral vectors containing neurotrophic factor genes primarily focus on the parameters of cell viability [37,49,50] or the volume of the site of stroke [51,52], or they are devoted to studying neurogenesis activation in the adult brain [38]. However, the question regarding the functional integrity of neural cells after virus vector administration and the influence of stress factors remain unexplored. 

Currently available evidence indicates that neural networks, rather than individual neurons, are the minimal functional unit of the CNS. Processes such as memory consolidation, transmission, and storage of information occur at this level of CNS organization [53,54]. Investigation of BDNF effects on the functional activity of neural networks in normal and stress conditions allows the identification of the protective mechanisms of BDNF on the network level.

Our studies demonstrate that application of AAV-Syn-BDNF-EGFP in normoxia does not change cell viability or the main parameters of spontaneous bioelectrical activity in primary hippocampal cultures. These data are of great interest because previous studies have demonstrated the effect of BDNF transient application on mature brain neural network activity [24,42]. It should be assumed that constant overexpression causes compensatory processes in neural cells and that transient effects are not commensurable with chronically increased concentration. The main functions of BDNF are mediated by its interaction with tropomyosin-related kinase B (TrkB) receptor, which could be also considered a potential therapeutic agent [55,56]. In this regard, investigations have demonstrated that increasing BDNF expression at the target sites leads to suppression of TrkB receptors, and thereby reduces the effect of BDNF chronic delivery over time. A sequence of genes encoding BDNF ligand and TrkB receptor in a single transgene was proposed to be more effective [57]. These data undoubtedly lay the foundation for future studies on the long-term effect of the developed virus vector on the expression of TrkB receptors. However, in the present study, we demonstrated the antihypoxic and neuroprotective effects of the developed virus vector.

Our data demonstrate that the application of AAV-Syn-BDNF-EGFP partially preserves the spontaneous bioelectrical activity of hippocampal neural networks in hypoxic conditions. Cross-correlation analysis of the electrophysiological data obtained from multi-electrode arrays demonstrated that acute hypoxia decreased the values of the spontaneous bioelectrical activity parameters and restructured and simplified the neural network functional structure by reducing the number of connections between individual electrodes and the number of hubs in the network. These changes are probably caused by death of a significant portion of functionally important neurons in neuronal networks, which is consistent with experiments conducted on cell culture viability.

AAV-Syn-BDNF-EGFP application prevented cell death under hypoxic influence and maintained the main parameters of spontaneous bioelectrical activity (the number of network bursts, the number of spikes in a burst) even in the distant posthypoxic period. A comparative analysis of the effects of BDNF overexpression and the use of recombinant BDNF showed similar results for both methods of neurotrophin administration.

BDNF overexpression maintains a comprehensive internal functional structure of neural networks in the posthypoxic period by preserving the number of connections composing a hub and stabilizing or increasing the number of hubs. Moreover, the activation pattern of a network burst did not significantly change, whereas in control cultures, the time of signal transmission between the network elements increased. This observation could be explained by the preservation of cell viability, as we also demonstrated. In addition, we found the ability of AAV-Syn-BDNF-EGFP to partially eliminate hypoxic effects exerted on the functional calcium activity in primary hippocampal cultures.

Several studies have recently demonstrated that the use of viral constructs carrying the *BDNF* gene stimulates neuronal growth. Data were obtained from a spinal cord traumatic model and evidence significant intensification of axons using both scaffolds with cell therapy and a single injection of the BDNF virus construct [40,58,59]. It can be assumed that similar molecular processes also occur during brain tissue damage; thus, BDNF possibly induces the formation of new synapses and restores the neural network functional structure.

Importantly, the effects of BDNF can be mediated by its influence on both neurons and astrocytes. Astrocytes are known to predominantly express the TrkB-T1 isoform, thus leading to PLC (Phospholipase C) activation and, consequently, to BDNF-dependent Ca^2+^ release [60]. Thus, astrocytes are able to regulate Ca^2+^ activity. Various reports demonstrated that TrkB-T1 controls morphology and cytoskeletal changes in astrocytes [61,62], regulates Rho GTPase activity [61], and may stimulate PLC and PKC (Protein kinase C) [60]. Astrocytes are currently regarded as an active component of the synapse (the third element of tripartite synapses) [63] that participates in the regulation of nerve impulse transmission. Astrocytic stimulation can lead to elevated intracellular Ca^2+^ and the regulation of neurotransmitter release (e.g., ATP, D-serine, and glutamate), controlling synaptic transmission and synaptic plasticity [64,65]. These findings indicate that astrocytes can play an important role in preventing neuronal functional disturbances under the influence of damaging factors.

Considering the potential applications of the developed construct, it should be mentioned that hypoxia is not only one of the most important factors of ischaemic damage, but also a component of the pathogenetic process of a number of other diseases, including neurodegenerative diseases. For instance, it has been hypothesized that capillary metabolism disturbance and hypoxia play important roles in the development of Alzheimer’s disease [66,67,68]. Microvascular dysfunction and chronic cerebral hypoperfusion are observed in metabolic syndrome [69].

Previously published studies have demonstrated the successful use of adenoviral constructs for BDNF overexpression in Alzheimer’s disease [70,71], Huntington’s disease [72], and Friedrich’s motor ataxia [39]. We believe that the present work expands our understanding of the molecular and cellular mechanisms underlying the positive effects of BDNF against neurodegeneration.

On the other hand, extensive experimental data point to the need to conduct further investigations of the effects of gene therapy with neurotrophins and the assessment of possible side effects. The application of AAV-BDNF in a rat model of Parkinson’s disease increased dyskinesia when the standard drug L-DOPA (L-3,4-dihydroxyphenylalanine) was used. The authors explained this finding by the maintenance of cell viability as well as the effects of BDNF on serotoninergic neurotransmission [73].

Thus, the AAV-Syn-BDNF-EGFP virus vector maintains brain cell viability and preserves the neural network functional structure in the late posthypoxic period.

## 4. Materials and Methods 

### 4.1. Ethics Statement

All experimental protocols in this study were approved by the Bioethics Committee ofthe Institute of Neuroscience, Lobachevsky State University , and experimental protocols were carried out according to Act708n (23 August 2010) of the Russian Federation National Ministry of Public Health, which states the rules of laboratory practice for the care and use of laboratory animals, and the Council Directive 2010/63 EU of the European Parliament (22 September 2010) on the protection of animals used for scientific purposes. C57BL6J mice were killed by cervical vertebra dislocation. Embryos were then surgically removed and sacrificed by decapitation.

### 4.2. Plasmids

We utilized AAV-Syn-EGFP, pDP5, DJvector, and pHelper to plasmids to develop the virus construct. The AAV-Syn-EGFP plasmid is derived from the bacterial pUC19 plasmid; it carries the sequences of the human synapsin (hSyn) promoter, the WPRE enhancer, and the SV40 polyA signal sequence flanked by the inverted terminal repeats (ITRs) from the adeno-associated serotype 2 virus (AAV2). The other three plasmids (pDP5, DJvector, and pHelper) are helper plasmids required to assemble the adeno-associated virus vector that carries the *Rep* and *Cap* genes.

The developed AAV-Syn-BDNF-EGFP included the following sequences: (1) the human synapsin (hSyn) promoter, allowing expression of the gene of interest only in neuronal cells; (2) the regulatory WPRE (woodchuck hepatitis posttranscriptional regulatory element) enhancer, which markedly strengthens hSyn function; (3) a multilinker for ORF (open reading frame) cloning of the embedded gene; (4) the *EGFP* gene; (5) the SV40 polyA signal sequence flanked by ITRs (inverted terminal repeats) from the AAV serotype 2; (6) a gene cassette encoding ampicillin resistance (AmpR promoter and AmpR gene) for positive selection of colonies carrying this plasmid; and (7) a sequence corresponding to the nucleotide sequence encoding the functional BDNF protein.

We designed primers, mBDNF-EcoRI-fw and mBDNF-BamHI-rv (5′-ATTGAATTCATGGGCCACATGCTGTCC-3′ and 5′-AATGGATCCAATCTTCCCCTTTTAATGGTCAGTG-3′), containing restriction sites for subsequent restriction and ligation into the constructed vector, temperature regimes, and the time of the reaction. The initial denaturation was conducted at 95 °C for 1 min. Then, the following working cycle was repeated 30 times: denaturation at 95 °C for 15 s, primer annealing at 65 °C for 10 s, and elongation at 72 °C for 40 s. Final chain completion was performed at 72 °C for 7 min. As a result, the coding sequence for the neurotrophic factor BDNF was obtained. 

### 4.3. Isolation of Total RNA

Isolation of total RNA from biological samples (mouse brain tissue) for subsequent production of cDNA encoding the BDNF protein was carried out according to the standard protocol using the Extract RNA reagent (Eurogen, Moscow, Russia).

### 4.4. Polymerase Chain Reaction (PCR) 

A commercial Phusion High-Fidelity PCR Kit containing Phusion High-Fidelity DNA polymerase (Thermo Fisher Scientific, Waltham, MA, USA) was used to perform PCR for the generation of cloned fragments of the *BDNF* gene. The advantage of this polymerase is its high activity and accuracy. Reverse transcription PCR was performed using Moloney murine leukaemia virus (MMLV) reverse transcriptase (Eurogen, Russia).

### 4.5. Plasmid Cloning

Purification of the resulting BDNF sequence was performed using a commercial Isolate II PCR and Gel Kit (Bioline, London, UK) according to the manufacturer’s protocol. For the construction of the plasmid, enzymes from Thermo Fisher Scientific (USA) and New England Biolabs (Ipswich, MA, USA) were used. Restriction of the cloned BDNF fragment and AAV-Syn-EGFP-kid2 plasmid was performed with EcoRI and BamHI restriction enzymes to form sticky ends (Figure 1). A ligase mixture was further used to transform competent *E. coli* TopTEN cells.

### 4.6. Transformation of E. coli Bacterial Cell Plasmid DNA, Generation and Isolation of Plasmid DNA

Competent cells of *E. coli* TopTEN were used for the transformation. The bacterial cells were kept on ice for 15–20 min, after which the cloned AAV-Syn-BDNF-EGFP plasmid was added in a volume of 1/10 of the cell volume, and the mixture was incubated for 20 min on ice. The cells were then subjected to heat shock at 42 °C for 45 s in a water bath and incubated on ice for 3 min. 

After a 30–90-min incubation at 37 °C under continuous shaking (200–300 rpm) in a three-fold volume of synthetic oil based (SOB) medium for the cultivation of prokaryotic cells, the transformed cells were plated on petri dishes containing Luria–Bertani (LB) medium, agar, and ampicillin (100 μg/mL). Cultivation on the plates was carried out at 37 °C for 12–16 h, after which some of the obtained bacterial colonies were further grown in liquid mini- and maxi-cultures. Then, plasmid DNA was isolated using commercial sets of ISOLATE II Plasmid Mini Kit (Bioline, London, UK) and NucleoBondXtra Midi/Maxi (MACHEREY-NAGEL, Berlin, Germany) according to the manufacturer’s protocol.

### 4.7. Cultivation of the Human Embryonic Kidney Cell Line HEK 293T and Transfection

The conditions for the cultivation of the human embryonic kidney (HEK) 293T cell line were identical to those indicated in the culture certificate: DMEM; 10% bovine embryonic serum; gentamicin 15 μg/mL. The passage procedure was as follows: cell removal with trypsin 0.25% and Versene 0.02% (1:2–1:3), seeding ratio of 1:2–1:3, and optimal density (3.0–5.0) × 10^4^ cells/cm^2^. Cultivation was carried out in a CO_2_ incubator at 37 °C, 5% CO_2_, and 80% humidity.

The shuttle plasmid AAV-Syn-BDNF-EGFP and the helper plasmids pDP5, DJvector, and pHelper were used for HEK transfection. The expression and helper plasmids were mixed in equimolar amounts (20 μg each) with DMEM. A mixture of polyethylenimine (PEI)/DMEM was added to the DNA/DMEM mixture and incubated for 15 min at room temperature, after which the volume of DMEM was adjusted to 15 mL. The HEK 293T cell culture medium was replaced with a mixture of PEI/DNA/DMEM for 4–5 h at 37 °C, 5% CO_2_. Then, the medium was completely replaced with DMEM containing 2% serum, and the cells were incubated for 3–5 days at 37 °C, 5% CO_2_.

### 4.8. Isolation and Purification of Adeno-Associated Virus Particles

After transfection, the HEK 293T cells were removed from the substrate using phosphate-buffered saline (PBS) containing no Ca^2+^ and no Mg^2+^. The cells were centrifuged at 1000 rpm for 5 min, and the precipitate dissolved in 2 mL of lysis buffer. The lysate was subjected to three freeze/thaw cycles (−80 °C). After the final thawing, the cell lysate was centrifuged at 2000 rpm for 5 min. Then, the lysate was enzymatically treated with benzonase to remove nucleotides and nucleic acids not protected by the virus capsids and centrifuged at 2000–2500 rpm for 5 min to sediment various protein granules and debris of large molecules. The supernatant was filtered through a 0.45 μm filter. Additional purification and concentration of the virus preparation was carried out using Amicon Ultra-15 columns (Merck Millipore, Burlington, MA, USA). 

### 4.9. Cultivation of Dissociated Hippocampal Cells

The following studies were conducted on cultures of dissociated hippocampal cells obtained from E18 embryos of C57BL/6 mice. We followed the basic rules for the maintenance and care of experimental animals according to the Rules for the Work using Experimental Animals (Russia, 2010) and the International Guiding Principles for Biomedical Research Involving Animals (CIOMS and ICLAS, 2012). The ethical principles established by the European Convention for the Protection of Vertebrate Animals used for Experimental and Other Scientific Purposes (Strasbourg, 2006) were also followed. The permission to carry out experimental studies on animals was obtained from the ethics committee of the Lobachevsky State University of Nizhni Novgorod, protocol 25 from 24 November 2017.

Embryonic hippocampal cells were enzymatically dissociated with a 0.25% trypsin solution (Gibco, Waltham, MA, USA). Primary cultures were cultured in Neurobasal™ medium (Thermo Fisher Scientific, USA) complexed with the B27 bioactive additive (Thermo Fisher Scientific, Waltham, MA, USA), L-glutamine (Thermo Fisher Scientific, USA), and foetal calf serum (PanEko, Moscow, Russia) according to the previously developed protocol [2] for 21 days on glasses coated with polyethyleneimine (Sigma, Burlington, MA, USA) to enhance the adhesion of cells to the surface. The initial density of the cultured cells on the matrix was 9000 cells/mm^2^. The cultures were maintained in a CO_2_ incubator (MCO-18AIC, Sanyo, Moriguchi, Osaka, Japan) at 35.5 °C and a gas mixture containing 5% CO_2_ [2].

To infect cultures, 3 μL of the viral preparation was mixed with 50 μL of fresh culture medium. The medium was temporarily removed from the culture dishes, and the working solution of the viral vector was directly added to the cells. The cups were placed in an incubator at 35.5 °C with 5% CO_2_ for 20 min, and then, the culture medium was returned to the cultures. Green fluorescent protein expression in the cultures was controlled daily from day 1 after infection using confocal microscopy.

### 4.10. Immunocytochemistry

Primary polyclonal chicken antibodies to BDNF and secondary polyclonal antibodies conjugated with goat anti-chicken IgY H & L fluorescent label (Alexa Fluor 647) (Abcam, Cambridge, UK) were used in the test. Cell cultures were fixed in a 4% solution of paraformaldehyde in PBS for 20 min at room temperature; a solution of 0.2% Triton X-100/PBS was used for cell permeabilization. The stained material was imaged using a Zeiss 510 NLO fluorescent confocal microscope (Carl Zeiss, Oberkochen, Germany).

To evaluate BDNF expression levels, immunocytochemical data were analysed using a custom ImageJ plugin. To conduct the data analysis, we compared culture samples with equal density and obtained images under the same laser power and photodetector settings. The average fluorescence intensity in the red channel, corresponding to the level of BDNF expression in an observation field, was estimated.

### 4.11. Electrophysiological Methods

An MED64 system (Alpha MED Science, Ibaraki, Osaka, Japan) and multi-electrode arrays with integrated 64 planar indium tinoxide (ITO) platinum black electrodes were used to simultaneously record extracellular action potentials. Microelectrode arrays (MEAs) were designed in an 8 × 8 array (64 total) with a 50 × 50 μm electrode size and 150 μm spacing. The sampling rate was 20 kHz/channel. All signal and statistical analyses were performed using custom software (MATLAB^®^6.0, Natick, MA, USA).

Small network bursts were detected by calculating the total spiking rate (TSR), which considered the total number of spikes from all electrodes within 50-ms time bins. The criterion of a small network burst was the rapid appearance of a large number of spikes over four electrodes within a small (50-ms) time bin [2,43].

### 4.12. Spike Detection

The recorded extracellular action potentials were detected by threshold calculations using the signal median:

(1) $$T=N_{S}\sigma ,\sigma =median(\frac{\left|x\right|}{0.6745})$$

where *x* is the bandpass-filtered (0.3–8 kHz) data signal, *σ* is an estimate of the standard deviation of the signal without spikes, and *N_S_* is a spike detection coefficient that determines the detection threshold. Threshold estimations based on the median of the signal in the form of Equation (1) are less dependent on the frequency of the spikes than threshold estimates based on the standard deviation during signal processing. The coefficient 0.6745 in Equation (1) was used to normalize the median of the absolute signal to the standard deviation. *N_S_* = 4 was used for all data, which allowed the reliable detection of spikes with amplitudes greater than 20 μV. The minimal interspike interval was set to 1 ms. Detected spikes were plotted using raster diagrams [2,43].

### 4.13. Small Burst Detection

We recorded spontaneous bursting activity to analyse the BDNF effect on the functional characteristics of the neural network in primary hippocampal cultures. The TSR was determined by counting the total number of spikes from all electrodes within 50-ms time bins for small network burst detection. The rapid emergence of a large number of spikes over multiple electrodes within a small (50-ms) time bin was used as the criterion for a small network burst. Spontaneous activity in the cultures corresponds to basal stochastic activity, which was observed in fractions of cells together with short bursting episodes. The spike trains (approximately one spike per 10–100 ms) are considered basal activity. To reveal bursts, we used threshold detection based on the statistical characteristics of the spontaneous activity TSR(t). The burst threshold was set to TBurst = 0.1 × σ TSR, where σ TSR is the standard deviation of TSR(t). The burst detection threshold coefficient was empirically set to 0.1, yielding the best estimate for the burst initiation and end points according to the raster diagram. Simulations of bursts with frequencies up to 5 Hz revealed that the estimated burst durations were within 10% of the actual values. Statistical analysis of the bursting activity characteristics was performed using analysis of variance (ANOVA) (*p* < 0.05) [2,43].

### 4.14. Cross-Correlation Method and Graphs

The dataset, obtained from spontaneous bioelectrical network activity recordings, was represented as a raster plot. The existence of functional connections between neuronal groups was not obviously based on visual analysis. The network graph method was then used to detect the neuronal groups.

First, to assess the degree of synchronization between all pairs of cells, taking into account axonal delays, the proportion of transmitted spikes was calculated. This measure is an analogue of the cross-correlation coefficient for the continuous signals. According to this method, the number of “delayed synchronous” spikes was calculated. These spikes must be recorded from both channels within a tolerance interval of δ, for which the time delay τ between the centres of the spikes is proportional to the distance between the electrodes. The number of delayed synchronous spikes was normalized by the number of spikes received by postsynaptic neuron, *n_j_*. Thus, the cross-correlation matrix was calculated by the following formula:

(2) $$N_{ij}=\frac{n_{synchr}}{n_{j}}$$

We then selected the largest 5% of *C_ij_* coefficients and defined a set of indices, that is, hubs of cells with a maximum number of functionally active connections. In addition, for each hub *i*, we calculated the number of connections to index *i* within the *C_ij_* array.

Next, the graph was constructed. The vertex size is proportional to the number of significant connections. The edge of the graph corresponds to the functional connections of spikes transferred from one neuron to another at individual time points for each pair of axonal delays, that is, τ ± *δ*/2 [45].

### 4.15. Ca^2+^ Imaging

To conduct functional calcium imaging, Oregon Green 488 BAPTA-1 AM (OGB-1) (0.4 μM, Invitrogen, O-6807, Waltham, MA, USA) was dissolved in dimethylsulfoxide (DMSO) (Sigma, D8418, Burlington, MA, USA) with 4% Pluronic F-127 (Invitrogen, P-3000MP, USA) and then added to the culture medium for 40 min. After incubation, to allow the OGB-1 molecules to be fully absorbed by the cells, the cells were washed for 15 min with dye-free medium. A confocal laser-scanning microscope (Zeiss LSM 510, Oberkochen, Germany) with a W Plan-Apochromat 20×/1.0 objective was used to visualize spontaneous calcium activity in the dissociated cultures.

Cytosolic Ca^2+^ was visualized via OGB-1 excitation with 488 nm Argon laser excitation and emission detection with a 500–530 nm filter. Time series of 256 × 256 pixel images of 420  × 420 μm fields of view were recorded at 4 Hz. A confocal pinhole of 1 airy unit was used to obtain an axial optical slice resolution of 1.6 μm.

Quantitative evaluation of Ca^2+^ transients was performed offline using custom-made software in C^++^ Builder. Cell regions from fluorescent images were manually selected. The Ca^2+^ fluorescence of each cell in each frame was calculated as the average fluorescence intensity (F, relative units from 0 to 255) of the pixels within the defined cell region. Single Ca^2+^ signals were identified using the following algorithm. First, each trace from all of the cells was filtered by averaging two neighbouring points in the sample set. Next, we calculated a simple derivative of the signal by determining the difference between each pair of consequent points. The pulses were identified from the derivative of the trace using a threshold detection algorithm. The threshold was estimated as the detection accuracy coefficient multiplied by the standard deviation of the derivative of the trace. Suprathreshold points on the derivative of the trace were taken as the beginnings and ends of the pulses [74].

### 4.16. Hypoxia Model

Hypoxia modelling was conducted by replacing the normoxic culture medium with a medium containing low oxygen (0.37 mL/L) for 10 min on day 14 of culture development in vitro (DIV), according to the previously described protocol [2,45].

### 4.17. Cell Viability Detection

For viability determination of dissociated hippocampal cells during the seven days of the posthypoxic period, we estimated the ratio of the number of dead cells stained by propidium iodide (Sigma, P4170, Germany) to the total number of cells stained by bisbenzimide (Invitrogen, H3570, USA). Propidium iodide and bisbenzimide at concentrations of 5 μg/mL and 1 μg/mL, respectively, were added to the culture medium 30 min before viability registration on days 1, 3, and 7 of the posthypoxic period [75]. Cells were observed using a Leica DMIL HC inverted fluorescence microscope (Leica, Wetzlar, Germany) with a 10×/0.2Ph1 objective.

### 4.18. Statistical Analysis

The obtained data are presented as the mean ± standard error of the mean (M ± SEM). The significance of the differences between the experimental groups was determined using the ANOVA package in SigmaPlot 11.0 (Systat Software Inc., San Jose, CA, USA). Differences were considered statistically significant at *p* < 0.05.

## 5. Conclusions

BDNF overexpression induced by AAV-Syn-BDNF-EGFP has neuroprotective and antihypoxic effects in an acute hypoxia model in vitro. Increased BDNF expression allows the maintenance of cell viability, partially supports the spontaneous bioelectrical and calcium activity of neural networks in primary hippocampal cultures, and preserves their functional structure. Notably, in normoxia, changes in spontaneous bioelectrical activity of neural networks under AAV-Syn-BDNF-EGFP were not detected.

## Figures and Tables

**Table 1:** Main parameters of spontaneous bioelectrical activity in primary hippocampal cell cultures.

Parameter	Group	8 DIV	14 DIV	21 DIV
number of small network bursts/10 min	Sham	56.6 ± 4.9	102.2 ± 18.6	186 ± 23.5
	AAV-Syn-BDNF-EGFP	63.2 ± 10.56	135.2 ± 24.8	235.5 ± 43.15
number of spikes per burst	Sham	224.2 ± 30.9	302.2 ± 18.6	483 ± 52.5
	AAV-Syn-BDNF-EGFP	183.2 ± 30.6	310.2 ± 20.8	438.5 ± 61.5

Analysis of variance (ANOVA), *n* = 24. DIV—day in vitro; AAV—adeno-associated virus; BDNF—brain-derived neurotrophic factor.

**Table 2:** Analysis of cell viability in primary hippocampal cultures during the posthypoxic period.

Number of Viable Cells, %	Sham	Hypoxia	Hypoxia + BDNF 1 ng/mL	Hypoxia + AAV-Syn-BDNF-EGFP
1st day after hypoxia	98.07 ± 0.58	91.23 ± 0.22 *	97.21 ± 0.43 #	96.28 ± 0.58 #
3rd day after hypoxia	95.34 ± 0.81	81.23 ± 4.34 *	87.21 ± 1.75 #	90.13 ± 0.99 *#
7th day after hypoxia	91.39 ± 1.34	70.46 ± 5.14 *	83.86 ± 4.57 #	91.75 ± 1.51 #

* versus “sham”, # versus “hypoxia”, *p* < 0.05, ANOVA, *n* = 36.

**Table 3:** The main parameters of spontaneous calcium activity in primary hippocampal cultures on day 7 after hypoxia modelling (28 DIV).

The Main Parameters of Spontaneous Calcium Activity	Sham	Hypoxia	Hypoxia + AAV-Syn-BDNF-EGFP
Number of cells that exhibited Ca^2+^ activity, %	97.66 ± 1.47%	35.58 ± 5.46% *	65.14 ± 5.27% *#
Duration of Са^2+^ oscillations, s	6.23 ± 0.16	7.17 ± 0.28 *	5.87 ± 0.9
Frequency of Са^2+^ oscillations, osc/min	6.9 ± 0.31	1.24 ± 0.134 *	5.84 ± 0.28 *#

*—versus “sham”, #—versus “hypoxia”, *p* < 0.05, ANOVA, *n* = 9.

## Supplementary Materials

Supplementary materials can be found at http://www.mdpi.com/1422-0067/19/8/2295/s1.

## References

[1] Neumann T.J., Thompson J.W., Raval A.P., Cohan C.H., Koronowski K.B., Perez-Pinzon M.A. (2015). Increased BDNF protein expression after ischemic or PKC epsilon preconditioning promotes electrophysiologic changes that lead to neuroprotection. J. Cereb. Blood Flow Metab..

[2] Vedunova M.V., Mishchenko T.A., Mitroshina E.V., Mukhina I.V. (2015). TrkB-Mediated Neuroprotective and Antihypoxic Properties of Brain-Derived Neurotrophic Factor. Oxid. Med. Cell. Longev..

[3] Huang W., Meng F., Cao J., Liu X., Zhang J., Li M. (2017). Neuroprotective Role of Exogenous Brain-Derived Neurotrophic Factor in Hypoxia-Hypoglycemia-Induced Hippocampal Neuron Injury via Regulating Trkb/MiR134 Signaling. J. Mol. Neurosci..

[4] Zhao H., Alam A., San C.Y., Eguchi S., Chen Q., Lian Q., Ma D. (2017). Molecular mechanisms of brain-derived neurotrophic factor in neuro-protection: Recent developments. Brain Res..

[5] Da Silva Meirelles L., Simon D., Regner A. (2017). Neurotrauma: The Crosstalk between Neurotrophins and Inflammation in the Acutely Injured Brain. Int. J. Mol. Sci..

[6] Harris N.M., Ritzel R., Mancini N.S., Jiang Y., Yi X., Manickam D.S., Banks W.A., Kabanov A.V., McCullough L.D., Verma R. (2016). Nano-particle delivery of brain derived neurotrophic factor after focal cerebral ischemia reduces tissue injury and enhances behavioral recovery. Pharmacol. Biochem. Behav..

[7] Ramos-Cejudo J., Gutiérrez-Fernández M., Otero-Ortega L., Rodríguez-Frutos B., Fuentes B., Vallejo-Cremades M.T., Hernanz T.N., Cerdán S., Díez-Tejedor E. (2015). Brain-derived neurotrophic factor administration mediated oligodendrocyte differentiation and myelin formation in subcortical ischemic stroke. Stroke.

[8] Schäbitz W.R., Schwab S., Spranger M., Hacke W. (1997). Intraventricular brain-derived neurotrophic factor reduces infarct size after focal cerebral ischemia in rats. J. Cereb. Blood Flow Metab..

[9] Ploughman M., Windle V., MacLellan C.L., White N., Doré J.J., Corbett D. (2009). Brain-derived neurotrophic factor contributes to recovery of skilled reaching after focal ischemia in rats. Stroke.

[10] Zhang X., Zhou Y., Li H., Wang R., Yang D., Li B., Fu J. (2018). Intravenous administration of DPSCs and BDNF improves neurological performance in rats with focal cerebral ischemia. Int. J. Mol. Med..

[11] Schäbitz W.R., Berger C., Kollmar R., Seitz M., Tanay E., Kiessling M., Schwab S., Sommer C. (2004). Effect of brain-derived neurotrophic factor treatment and forced arm use on functional motor recovery after small cortical ischemia. Stroke.

[12] Berretta A., Tzeng Y.C., Clarkson A.N. (2014). Post-stroke recovery: The role of activity-dependent release of brain-derived neurotrophic factor. Expert Rev. Neurother..

[13] Zhu J.M., Zhao Y.Y., Chen S.D., Zhang W.H., Lou L., Jin X. (2011). Functional recovery after transplantation of neural stem cells modified by brain-derived neurotrophic factor in rats with cerebral ischaemia. J. Int. Med. Res..

[14] Phillips H.S., Hains J.M., Armanini M., Laramee G.R., Johnson S.A., Winslow J.W. (1991). BDNF mRNA is decreased in the hippocampus of individuals with Alzheimer’s disease. Neuron.

[15] Peng S., Wuu J., Mufson E.J., Fahnestock M. (2005). Precursor form of brain-derived neurotrophic factor and mature brain-derived neurotrophic factor are decreased in the pre-clinical stages of Alzheimer’s disease. J. Neurochem..

[16] Iulita M.F., Millón M.B., Pentz R., Aguilar L.F., Do Carmo S., Allard S., Michalski B., Wilson E.N., Ducatenzeiler A., Bruno M.A. (2017). Differential deregulation of NGF and BDNF neurotrophins in a transgenic rat model of Alzheimer’s disease. Neurobiol. Dis..

[17] Arancibia S., Silhol M., Moulière F., Meffre J., Höllinger I., Maurice T., Tapia-Arancibia L. (2008). Protective effect of BDNF against beta-amyloid induced neurotoxicity in vitro and in vivo in rats. Neurobiol. Dis..

[18] Song J.H., Yu J.T., Tan L. (2015). Brain-Derived Neurotrophic Factor in Alzheimer’s Disease: Risk, Mechanisms, and Therapy. Mol. Neurobiol..

[19] Criscuolo C., Fabiani C., Bonadonna C., Origlia N., Domenici L. (2015). BDNF prevents amyloid-dependent impairment of LTP in the entorhinal cortex by attenuating p38 MAPK phosphorylation. Neurobiol. Aging.

[20] Tome D., Fonseca C.P., Campos F.L., Baltazar G. (2017). Role of Neurotrophic Factors in Parkinson’s Disease. Curr. Pharm. Des..

[21] Nam J.H., Leem E., Jeon M.T., Jeong K.H., Park J.W., Jung U.J., Kholodilov N., Burke R.E., Jin B.K., Kim S.R. (2015). Induction of GDNF and BDNF by hRheb(S16H) transduction of SNpc neurons: Neuroprotective mechanisms of hRheb(S16H) in a model of Parkinson’s disease. Mol. Neurobiol..

[22] Douglas-Escobar M., Rossignol C., Steindler D., Zheng T., Weiss M.D. (2012). Neurotrophin-induced migration and neuronal differentiation of multipotent astrocytic stem cells in vitro. PLoS ONE.

[23] Skaper S.D. (2018). Neurotrophic Factors: An Overview. Methods Mol. Biol..

[24] Rose C.R., Blum R., Kafitz K.W., Kovalchuk Y., Konnerth A. (2004). From modulator to mediator: Rapid effects of BDNF on ion channels. Bioessays.

[25] Martin J.L., Finsterwald C. (2011). Cooperation between BDNF and glutamate in the regulation of synaptic transmission and neuronal development. Commun. Integr. Biol..

[26] Kowiański P., Lietzau G., Czuba E., Waśkow M., Steliga A., Moryś J. (2018). BDNF: A Key Factor with Multipotent Impact on Brain Signaling and Synaptic Plasticity. Cell. Mol. Neurobiol..

[27] Schäbitz W.R., Steigleder T., Cooper-Kuhn C.M., Schwab S., Sommer C., Schneider A., Kuhn H.G. (2007). Intravenous brain-derived neurotrophic factor enhances poststroke sensorimotor recovery and stimulates neurogenesis. Stroke.

[28] Grade S., Weng Y.C., Snapyan M., Kriz J., Malva J.O., Saghatelyan A. (2013). Brain-derived neurotrophic factor promotes vasculature-associated migration of neuronal precursors toward the ischemic striatum. PLoS ONE.

[29] Cook D.J., Nguyen C., Chun H.N., Llorente I., Chiu A.S., Machnicki M., Zarembinski T.I. (2017). Carmichael ST. Hydrogel-delivered brain-derived neurotrophic factor promotes tissue repair and recovery after stroke. J. Cereb. Blood Flow Metab..

[30] Destot-Wong K.D., Liang K., Gupta S.K., Favrais G., Schwendimann L., Pansiot J., Baud O., Spedding M., Lelièvre V., Mani S. (2009). The AMPA receptor positive allosteric modulator, S18986, is neuroprotective against neonatal excitotoxic and inflammatory brain damage through BDNF synthesis. Neuropharmacology.

[31] Parnpiansil P., Jutapakdeegul N., Chentanez T., Kotchabhakdi N. (2003). Exercise during pregnancy increases hippocampal brain-derived neurotrophic factor mRNA expression and spatial learning in neonatal rat pup. Neurosci. Lett..

[32] Ahn S.Y., Chang Y.S., Sung D.K., Sung S.I., Ahn J.Y., Park W.S. (2017). Pivotal Role of Brain-Derived Neurotrophic Factor Secreted by Mesenchymal Stem Cells in Severe Intraventricular Hemorrhage in Newborn Rats. Cell Transplant..

[33] Angelova A., Angelov B., Drechsler M., Lesieur S. (2013). Neurotrophin delivery using nanotechnology. Drug Discov. Today.

[34] Angelov B., Angelova A., Filippov S.K., Drechsler M., Štěpánek P., Lesieur S. (2014). Multicompartment lipid cubic nanoparticles with high protein upload: Millisecond dynamics of formation. ACS Nano.

[35] Angelova A., Angelov B. (2017). Dual and multi-drug delivery nanoparticles towards neuronal survival and synaptic repair. Neural Regen. Res..

[36] Géral C., Angelova A., Lesieur S. (2013). From molecular to nanotechnology strategies for delivery of neurotrophins: Emphasis on brain-derived neurotrophic factor (BDNF). Pharmaceutics.

[37] LeVaillant C.J., Sharma A., Muhling J., Wheeler L.P., Cozens G.S., Hellström M., Rodger J., Harvey A.R. (2016). Significant changes in endogenous retinal gene expression assessed 1 year after a single intraocular injection of AAV-CNTF or AAV-BDNF. Mol. Ther. Methods Clin. Dev..

[38] Yu S.J., Tseng K.Y., Shen H., Harvey B.K., Airavaara M., Wang Y. (2013). Local administration of AAV-BDNF to subventricular zone induces functional recovery in stroke rats. PLoS ONE.

[39] Katsu-Jiménez Y., Loría F., Corona J.C., Díaz-Nido J. (2016). Gene Transfer of Brain-derived Neurotrophic Factor (BDNF) Prevents Neurodegeneration Triggered by FXN Deficiency. Mol. Ther..

[40] Liu S., Sandner B., Schackel T., Nicholson L., Chtarto A., Tenenbaum L., Puttagunta R., Müller R., Weidner N., Blesch A. (2017). Regulated viral BDNF delivery in combination with Schwann cells promotes axonal regeneration through capillary alginate hydrogels after spinal cord injury. Acta Biomater..

[41] Zhang J., Yu Z., Yu Z., Yang Z., Zhao H., Liu L., Zhao J. (2011). rAAV-mediated delivery of brain-derived neurotrophic factor promotes neurite outgrowth and protects neurodegeneration in focal ischemic model. Int. J. Clin. Exp. Pathol..

[42] Mishchenko T.A., Vedunova M.V., Mitroshina E.V., Pimashkin A.S., Mukhina I.V. (2015). Neurotropic Effect of Brain-Derived Neurotrophic Factor at Different Stages of Dissociated Hippocampal Cultures Development in vitro. Sovremennye Tehnologii v Medicine.

[43] Mukhina I.V., Kazantsev V.B., Khaspeckov L.G., Zakharov Y.N., Vedunova M.V., Mitroshina E.V., Korotchenko S.A., Koryagina E.A. (2009). Multielectrode matrices—New possibilities in investigation of the neuronal network plasticity. Sovremennye Tehnol. Medicine.

[44] Zhong J.B., Li X., Zhong S.M., Liu J.D., Chen C.B., Wu X.Y. (2017). Knockdown of long noncoding antisense RNA brain-derived neurotrophic factor attenuates hypoxia/reoxygenation-induced nerve cell apoptosis through the BDNF-TrkB-PI3K/Akt signaling pathway. Neuroreport.

[45] Shishkina T.V., Mishchenko T.A., Mitroshina E.V., Shirokova O.M., Pimashkin A.S., Kastalskiy I.A., Mukhina I.V., Kazantsev V.B., Vedunova M.V. (2018). Glial cell line-derived neurotrophic factor (GDNF) counteracts hypoxic damage to hippocampal neural network function in vitro. Brain Res..

[46] Shirokova О.М., Frumkina L.Е., Vedunova М.V., Mitroshina Е.V., Zakharov Y.N., Khaspekov L.G., Mukhina I.V. (2013). Morphofunctional Patterns of Neuronal Network Developing in Dissociated Hippocampal Cell Cultures. Sovremennye Tehnologii v Medicine.

[47] Chen A., Xiong L.-J., Tong Y., Mao M. (2013). The neuroprotective roles of BDNF in hypoxic ischemic brain injury. Biomed. Rep..

[48] Sun X., Zhou H., Luo X., Li S., Yu D., Hua J., Mu D., Mao M. (2008). Neuroprotection of brain-derived neurotrophic factor against hypoxic injury in vitro requires activation of extracellular signal-regulated kinase and phosphatidylinositol 3-kinase. Int. J. Dev. Neurosci..

[49] Liu Z., Ma D., Feng G., Ma Y., Hu H. (2007). Recombinant AAV-mediated expression of human BDNF protects neurons against cell apoptosis in Abeta-induced neuronal damage model. J. Huazhong Univ. Sci. Technol. Sci..

[50] Nakajima H., Uchida K., Yayama T., Kobayashi S., Guerrero A.R., Furukawa S., Baba H. (2010). Targeted retrograde gene delivery of brain-derived neurotrophic factor suppresses apoptosis of neurons and oligodendroglia after spinal cord injury in rats. Spine.

[51] Shi Q., Zhang P., Zhang J., Chen X., Lu H., Tian Y., Parker T.L., Liu Y. (2009). Adenovirus-mediated brain-derived neurotrophic factor expression regulated by hypoxia response element protects brain from injury of transient middle cerebral artery occlusion in mice. Neurosci. Lett..

[52] Tao J., Ji F., Liu B., Wang F., Dong F., Zhu Y. (2012). Improvement of deficits by transplantation of lentiviral vector-modified human amniotic mesenchymal cells after cerebral ischemia in rats. Brain Res..

[53] Yuste R. (2015). From the neuron doctrine to neural networks. Nat. Rev. Neurosc..

[54] Tong M.T., Peace S.T., Cleland T.A. (2014). Properties and mechanisms of olfactory learning and memory. Front. Behav. Neurosci..

[55] Guerzoni L.P., Nicolas V., Angelova A. (2017). In Vitro Modulation of TrkB Receptor Signaling upon Sequential Delivery of Curcumin-DHA Loaded Carriers Towards Promoting Neuronal Survival. Pharm. Res..

[56] Angelov B., Angelova A. (2017). Nanoscale clustering of the neurotrophin receptor TrkB revealed by super-resolution STED microscopy. Nanoscale.

[57] Osborne A., Wang A.X., Tassoni A., Widdowson P.S., Martin K.R. (2018). Design of a Novel Gene Therapy Construct to Achieve Sustained Brain-Derived Neurotrophic Factor Signaling in Neurons. Hum. Gene Ther..

[58] Gao M., Lu P., Lynam D., Bednark B., Campana W.M., Sakamoto J., Tuszynski M. (2016). *BDNF* gene delivery within and beyond templated agarose multi-channel guidance scaffolds enhances peripheral nerve regeneration. J. Neural Eng..

[59] Ziemlińska E., Kügler S., Schachner M., Wewiór I., Czarkowska-Bauch J., Skup M. (2014). Overexpression of BDNF increases excitability of the lumbar spinal network and leads to robust early locomotor recovery in completely spinalized rats. PLoS ONE.

[60] Rose C.R., Blum R., Pichler B., Lepier A., Kafitz K.W., Konnerth A. (2003). Truncated TrkB-T1 mediates neurotrophin-evoked calcium signalling in glia cells. Nature.

[61] Ohira K., Kumanogoh H., Sahara Y., Homma K.J., Hirai H., Nakamura S., Hayashi M. (2005). A truncated tropomyosin-related kinase B receptor, T1, regulates glial cell morphology via Rho GDP dissociation inhibitor 1. J. Neurosci..

[62] Ohira K., Funatsu N., Homma K.J., Sahara Y., Hayashi M., Kaneko T., Nakamura S. (2007). Truncated TrkB-T1 regulates the morphology of neocortical layer I astrocytes in adult rat brain slices. Eur. J. Neurosci..

[63] Perea G., Navarrete M., Araque A. (2009). Tripartite synapses: Astrocytes process and control synaptic information. Trends Neurosci..

[64] Bezzi P., Volterra A. (2001). A neuron-glia signalling network in the active brain. Curr. Opin. Neurobiol..

[65] Hamilton N.B., Attwell D. (2010). Do astrocytes really exocytose neurotransmitters?. Nat. Rev. Neurosci..

[66] Zhang F., Zhong R., Qi H., Li S., Cheng C., Liu X., Liu Y., Le W. (2018). Impacts of Acute Hypoxia on Alzheimer’s Disease-Like Pathologies in APPswe/PS1dE9 Mice and Their Wild Type Littermates. Front. Neurosci..

[67] Nucera A., Hachinski V. (2018). Cerebrovascular and Alzheimer disease: Fellow travelers or partners in crime?. J. Neurochem..

[68] Nielsen R.B., Egefjord L., Angleys H., Mouridsen K., Gejl M., Møller A., Brock B., Brændgaard H., Gottrup H., Rungby J. (2017). Capillary dysfunction is associated with symptom severity and neurodegeneration in Alzheimer’s disease. Alzheimer’s Dement..

[69] Herrera M.I., Udovin L.D., Toro-Urrego N., Kusnier C.F., Luaces J.P., Otero-Losada M., Capani F. (2018). Neuroprotection Targeting Protein Misfolding on Chronic Cerebral Hypoperfusion in the Context of Metabolic Syndrome. Front. Neurosci..

[70] Iwasaki Y., Negishim T., Inoue M., Tashiro T., Tabira T., Kimura N. (2012). Sendai virus vector-mediated brain-derived neurotrophic factor expression ameliorates memory deficits and synaptic degeneration in a transgenic mouse model of Alzheimer’s disease. J. Neurosci. Res..

[71] Jiao S.S., Shen L.L., Zhu C., Bu X.L., Liu Y.H., Liu C.H., Yao X.Q., Zhang L.L., Zhou H.D., Walker D.G. (2016). Brain-derived neurotrophic factor protects against tau-related neurodegeneration of Alzheimer’s disease. Transl. Psychiatry.

[72] Savolainen M., Emerich D., Kordower J.H. (2018). Disease Modification through Trophic Factor Delivery. Methods Mol. Biol..

[73] Tronci E., Napolitano F., Muñoz A., Fidalgo C., Rossi F., Björklund A., Usiello A., Carta M. (2017). BDNF over-expression induces striatal serotonin fiber sprouting and increases the susceptibility to l-DOPA-induced dyskinesia in 6-OHDA-lesioned rats. Exp. Neurol..

[74] Zakharov Y.N., Mitroshina E.V., Shirokova O., Mukhina I.V. (2013). Calcium transient imaging as tool for neuronal and glial network interaction study. Springer Proc. Math. Stat. Model. Algorithms Technol. Netw. Anal..

[75] Zhao Z., Lu R., Zhang B., Shen J., Yang L., Xiao S., Liu J., Suo W.Z. (2012). Differentiation of HT22 neurons induces expression of NMDA receptor that mediates homocysteine cytotoxicity. Neurol. Res..

