# Supplementary material for: AAV-Syn-BDNF-EGFP Virus Construct Exerts Neuroprotective Action on the Hippocampal Neural Network during Hypoxia In Vitro
Source: Int J Mol Sci. 2018 Aug 5;19(8):2295. doi: 10.3390/ijms19082295 (PMC6121472; doi:10.3390/ijms19082295)
Supplement: Supplementary file 1 [file ijms-19-02295-s001.pdf]

International Journal of
Molecular Sciences

MDPI

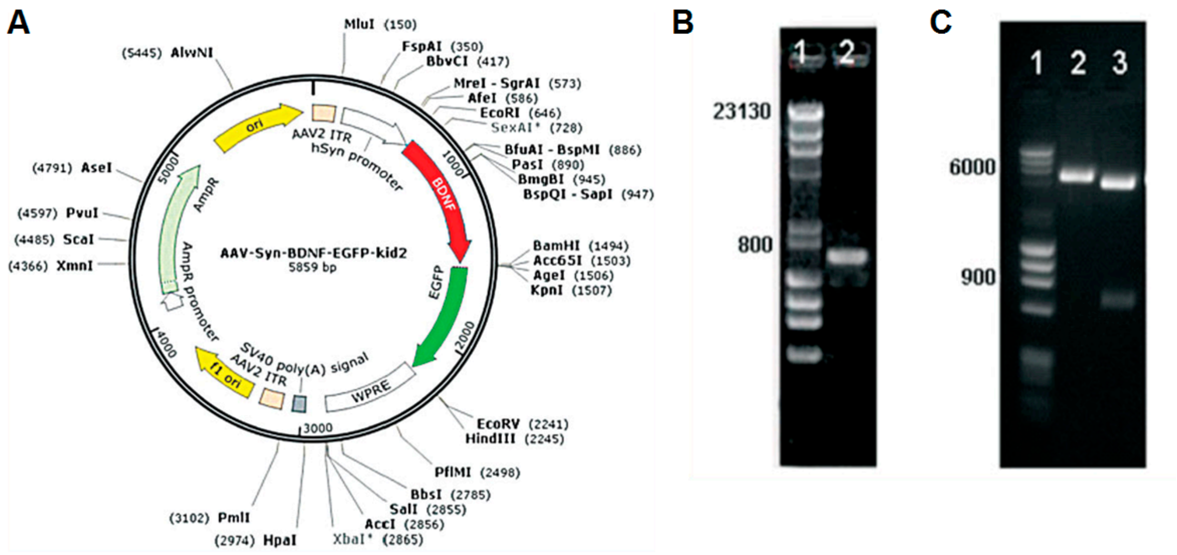

Figure S1. AAV-Syn-BDNF-EGFP-kid2 plasmid map constructed in SnapGene Viewer 3.1.2; **B** and **C** - qualitative analysis of PCR data **(B)** and restriction **(C)** by horizontal electrophoresis in agarose gel. **1.** – Marker of the DNA length; **2.** – AAV-Syn-BDNF-EGFP restriction by EcoRI enzyme, **3.** - AAV-Syn-BDNF-EGFP restriction by EcoRI and BamHI enzymes.

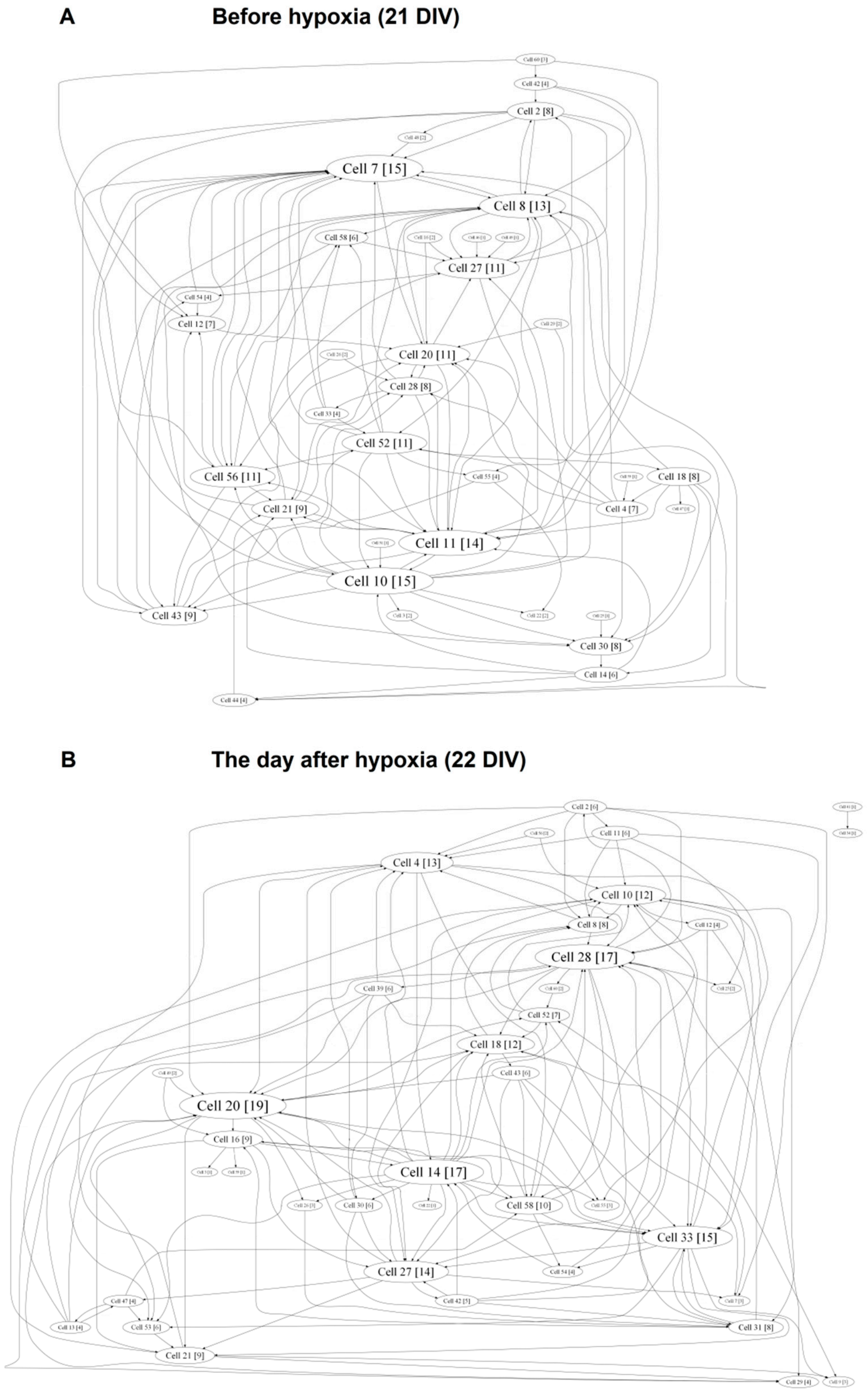

Figure S2. Internal functional structure of neural networks in the primary hippocampal cultures in hypoxia modelling. Graphical representation of the correlated connections among neurons in the network. The electrode number is presented as "Cell X". The number of connections on the electrode is indicated in square brackets. The vertex size is proportional to the number of significant connections. A – BDNF1 ng/ml before hypoxia (21 DIV), B – BDNF 1 ng/ml the day after hypoxia.

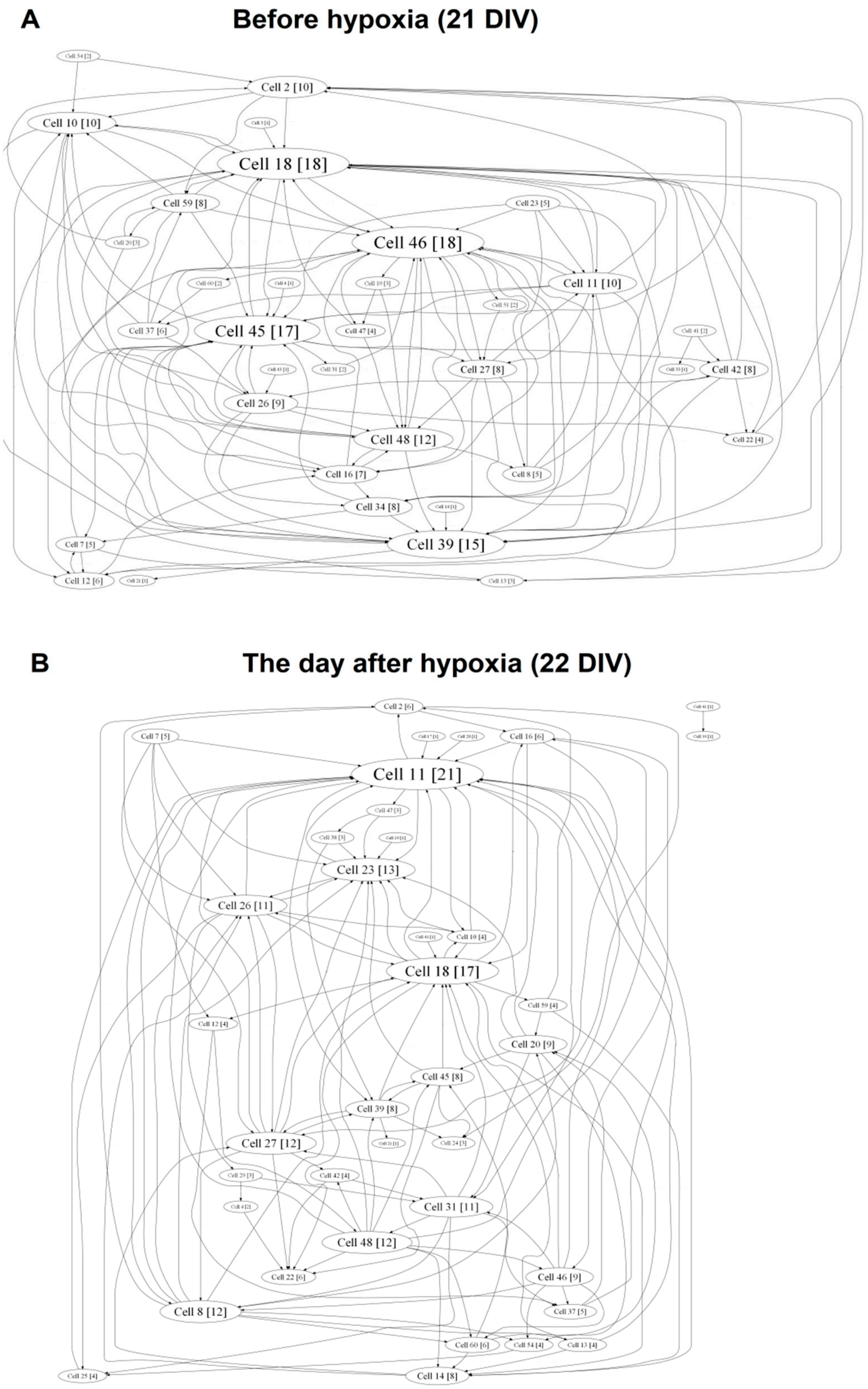

Figure S3. Internal functional structure of neural networks in the primary hippocampal cultures in hypoxia modelling. Graphical representation of the correlated connections among neurons in the network. The electrode number is presented as "Cell X". The number of connections on the electrode is indicated in square brackets. The vertex size is proportional to the number of significant connections. A - AAV-Syn-BDNF-EGFP before hypoxia (21 DIV), B – AAV-Syn-BDNF-EGFP the day after hypoxia.
